# Cell cycle alterations induced by urban PM2.5 in bronchial epithelial cells: characterization of the process and possible mechanisms involved

**DOI:** 10.1186/1743-8977-10-63

**Published:** 2013-12-19

**Authors:** Eleonora Longhin, Jørn A Holme, Kristine B Gutzkow, Volker M Arlt, Jill E Kucab, Marina Camatini, Maurizio Gualtieri

**Affiliations:** 1 Department of Environmental Sciences, POLARIS Research Centre, University Milano-Bicocca, Piazza della Scienza 1, 20126 Milano, Italy; 2 Division of Environmental Medicine, Norwegian Institute of Public Health, P.O. Box 4404, Nydalen N-0403 Oslo, Norway; 3 Analytical and Environmental Sciences Division, MRC-PHE-Centre for Environment and Health, King’s College London, 150 Stamford Street, London, SE1 9NH, UK

**Keywords:** PM2.5, BEAS-2B, Mitotic arrest, CYP enzymes, ROS

## Abstract

**Background:**

This study explores and characterizes cell cycle alterations induced by urban PM2.5 in the human epithelial cell line BEAS-2B, and elucidates possible mechanisms involved.

**Methods:**

The cells were exposed to a low dose (7.5 μg/cm^2^) of Milan winter PM2.5 for different time points, and the cell cycle progression was analyzed by fluorescent microscopy and flow cytometry. Activation of proteins involved in cell cycle control was investigated by Western blotting and DNA damage by ^32^P-postlabelling, immunostaining and comet assay. The formation of reactive oxygen species (ROS) was quantified by flow cytometry. The role of PM organic fraction versus washed PM on the cell cycle alterations was also examined. Finally, the molecular pathways activated were further examined using specific inhibitors.

**Results:**

Winter PM2.5 induced marked cell cycle alteration already after 3 h of exposure, represented by an increased number of cells (transient arrest) in G2. This effect was associated with an increased phosphorylation of Chk2, while no changes in p53 phosphorylation were observed at this time point. The increase in G2 was followed by a transient arrest in the metaphase/anaphase transition point (10 h), which was associated with the presence of severe mitotic spindle aberrations. The metaphase/anaphase delay was apparently followed by mitotic slippage at 24 h, resulting in an increased number of tetraploid G1 cells and cells with micronuclei (MN), and by apoptosis at 40 h. Winter PM2.5 increased the level of ROS at 2 h and DNA damage (8-oxodG, single- and double stand breaks) was detected after 3 h of exposure. The PM organic fraction caused a similar G2/M arrest and augmented ROS formation, while washed PM had no such effects. DNA adducts were detected after 24 h. Both PM-induced DNA damage and G2 arrest were inhibited by the addition of antioxidants and α-naphthoflavone, suggesting the involvement of ROS and reactive electrophilic metabolites formed via a P450-dependent reaction.

**Conclusions:**

Milan winter PM2.5 rapidly induces severe cell cycle alterations, resulting in increased frequency of cells with double nuclei and MN. This effect is related to the metabolic activation of PM2.5 organic chemicals, which cause damages to DNA and spindle apparatus.

## Background

In October 2013 the International Agency for Research on Cancer (IARC) classified outdoor air pollution as *carcinogenic to humans* (Group 1) [1]. Particulate matter (PM) is a well-known air pollutant and its adverse effects on human health are well established [2,3]. Increased levels of PM have been associated with exacerbation of airways disease in patients with asthma and Chronic Obstructive Pulmonary Disease (COPD) [4]. There is growing evidence linking long-term exposure to the fine PM fraction (PM2.5; aerodynamic diameter ≤ 2.5 μm) with increased risk of cardiovascular mortality [5,6] and lung cancer [7,8]. However, the understanding of the mechanisms by which PM exerts its various adverse effects is still incomplete and detailed *in vitro* studies are highly needed.

Urban air PM is a heterogeneous mixture of various types of particles originating from different sources. Combustion particles emitted from vehicles consist mainly of spherical primary carbon particles with diameters ranging from 20 to 30 nm, which tend to aggregate in PM1 and PM2.5 [9,10]. The small diameters of the primary carbon particles provide a relatively high surface area per mass unit, which facilitates the adsorption of various components to the particles, including metals, organic compounds and biological components like bacterial endotoxins [11,12]. In contrast, larger size particles as PM10 often are found to be arbitrarily-shaped mineral particles from road wear and soil dusts [13]. The composition of urban air PM also varies with season, and all these variables have a primary role in the promotion of the biological effects. This is evidenced by *in vitro* studies showing that, depending on composition, PM can trigger release of inflammatory mediators including various cytokines and chemokines [11,14], genotoxic effects [15-17] and cell death [11,18].

*In vitro* studies have demonstrated that PM may inhibit cell growth, by reducing proliferation and/or causing cell death [19-21]. The reduced proliferation has been linked to an arrest in various steps of the cell cycle [20-23]. Cell cycle progression can be blocked and/or delayed in response to various genotoxic stresses, but also to structural dysfunctions of various proteins. DNA-integrity checkpoints G1/S, G2/M and metaphase-anaphase (M/A) transition determine delays of the cell cycle [24,25]. The protein kinases ATM (ataxia telangiectasia mutated) and ATR (ATM and Rad3 related) contribute to the DNA damage response and activate the checkpoint protein kinases Chk1/2, which may result in cell cycle arrest by a p53-dependent or -independent pathway [26]. Both of these pathways regulate the activity of G1/S or G2/M transition promoters cyclin-dependent kinase (Cdk)/cyclin, such as Cdk1/cyclin B1, which drives the progression from G2 to the mitotic phase [26,27]. In the p53-dependent pathway, Chk1/2 phosphorylates p53 (Ser 15) which, through the transcriptional activation of downstream mediators p21 and 14-3-3, inhibits Cdk1/cyclin B1. In the p53-independent pathway, Chk1/2 phosphorylates Cdc25 and Wee-1, which cooperatively reduce Cdk1/cyclin B1 activity, leading to G2 arrest and preventing entry into mitosis [28].

The passage from metaphase to anaphase (M/A transition point) requires the disassembling of the Cdk1/cyclin B1 complex. The anaphase-promoting complex (APC) is responsible for the ubiquitination and subsequent degradation of cyclin B1 [29]. The spindle assembly checkpoint (SAC) acts on the mitosis delay at the M/A transition point, preventing the activation of APC until the mitotic spindle is correctly formed [26,30]. The inhibition of APC by SAC results in the stabilization of cyclin B1, which prevents the anaphase onset and karyokinesis until all chromosomes are properly attached to the bipolar mitotic spindle [29,31]. If the spindle is not properly attached to the chromosomes within a defined time period, the cell may enter a death process or may exit from mitosis without dividing the genetic material, a process named mitotic slippage. Cell death during mitosis or after mitotic slippage is termed mitotic catastrophe, an atypical mode of cell death, which often is due to premature or inappropriate entry into mitosis [29]. An abnormal spindle structure can be a consequence of DNA damage or can be directly originated by spindle-poisons. Thus, the identification of the specific stage at which a particular agent inhibits cell cycle progression, through the G1/S, G2/M or M/A transition points, has a pivotal role in the understanding of the mechanisms as well the final outcome.

Recently we have observed that exposure to 25 μg/cm^2^ of Milan winter PM2.5 for 20 h induced a mitotic arrest resulting in cell death by apoptosis in human bronchial epithelial cells (BEAS-2B) [21]. Effects involved in DNA-damage response, such as γH2AX and Chk2 over-expression, were detected at the low doses 5 and 7.5 μg/cm^2^. A further characterization of PM-induced cell cycle and mitotic alterations is important when trying to explain PM-induced chromosomal alterations, as well as its association with an increased risk of lung cancer [1,7,8].

In the present study, the effects of Milan winter PM2.5 on the cell cycle progression were characterized using the low dose 7.5 μg/cm^2^. This dose rapidly induced a delay in G2 phase, which was followed by a specific arrest at the M/A transition point and by an increased number of cells with double nuclei and micronuclei (MN). The proteins controlling the cell cycle process were investigated by Western blotting and the presence of mitotic spindle aberrations by fluorescence microscopy. The PM organic fraction and washed PM were tested to explore their role in the induced alterations. We further measured the formation of reactive oxygen species (ROS) and possible damage to the mitochondria and DNA. Finally, antioxidants and the AhR/CYP enzymes inhibitor alpha-naphthoflavone (α-NF) were used to investigate the importance of ROS and/or P450-catalyzed metabolites for PM-induced cell cycle alterations.

Our results indicate that the observed effects were associated with chemicals in the PM organic fraction. Using inhibitors and antioxidants, we showed that these compounds were activated via CYP enzymes to reactive electrophilic and/or radical metabolites which induced DNA damage and likely affected the chromosomal spindle apparatus.

## Results

### Cell cycle alterations in cells exposed to winter PM2.5

**Figure 1:**
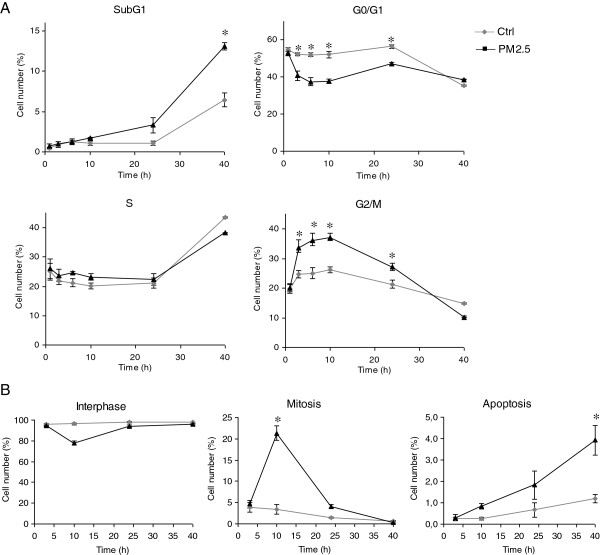
**Cell cycle analysis. (A)** Cell cycle of BEAS-2B cells exposed to 7.5 μg/cm^2^ of winter PM2.5 for 1, 3, 6, 10, 24 and 40 h. The results are representative of 4 independent experiments. **(B)** Mitotic cells scoring: BEAS-2B cells, exposed for 3, 10, 24 and 40 h to 7.5 μg/cm^2^ of winter PM2.5, were stained for DNA and β-tubulin and scored as interphasic, mitotic or apoptotic cells. The results are representative of 3 independent experiments; in each experiment 500 cells were scored. * Statistically significant difference from untreated cells (control), P < 0.05.

In preliminary studies we found that Milan winter PM2.5 induced a slight decrease in BEAS-2B cell proliferation, evidenced by microscopic observations, but no significant cell death (Figure 1A). To examine if the reduced proliferation was due to cell cycle alterations and consequent accumulation of cells at a specific cell cycle phase, cells were analysed at different time points by flow cytometry. Figure 1A illustrates an increase in the number of G2/M cells in the time interval from 3 to 24 h. After 3 h of PM-treatment, the number of G2/M cells was 33.5% compared to 24.7% in controls. The relative distribution of cells returned to the control values after 40 h of exposure. At this time point, a significant increase of subG1 cells (13.2% in PM exposed samples versus 6.5% in controls), representing cells with DNA < 2 N (possibly apoptotic and apoptotic/necrotic cells), was observed (Figure 1A).

In order to further characterize the G2/M arrest, and the subsequent subG1 increase, the amount of mitotic and apoptotic cells was screened by fluorescence microscopy at 3, 10, 24 and 40 h of exposure. Cells were stained for DNA and β-tubulin and scored according to nucleus and spindle morphology as interphasic, mitotic or apoptotic. At 3 h, in PM-treated samples the relative amount of mitotic cells (with evident chromosome condensation) was similar to controls (Figure 1B), suggesting that the G2/M increase was due to an accumulation of cells at the G2/M checkpoint. However, at 10 h a dramatic increase in the relative number of mitotic cells was observed (21.3% in treated cells versus 3.3% in controls). Interestingly, after 24 h the percentage of mitotic cells in exposed samples returned to control levels, without any marked change in the relative amount of necrotic and/or apoptotic cells until 40 h of treatment (Figure 1A and B), when a significant increase in apoptotic cells was observed.

### Cell cycle control

The mechanism leading to cell cycle alterations was investigated by analysing the expression and phosphorylation (activation) of two key proteins, p53 and Chk2, involved in the control of G2 checkpoint activation [26].

**Figure 2:**
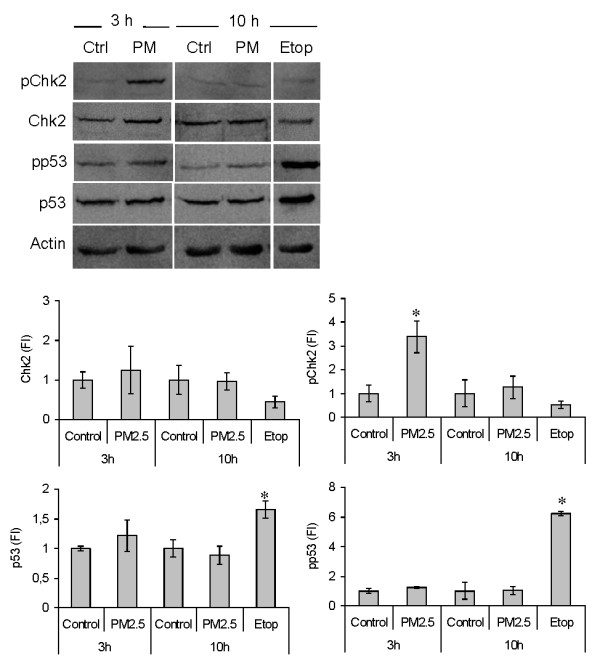
**Cell cycle control proteins expression.** The expression of p53/pp53 and Chk2/pChk2, proteins possibly involved in G2 checkpoint, was measured by Western blotting in BEAS-2B cells exposed for 3 and 10 h to 7.5 μg/cm^2^ of winter PM2.5. The topoisomerase II inhibitor etoposide (Etop, 250 nM, 10 h exposure) was used as positive control for p53/pp53. Representative images of the Western blotting are shown and the results of 3 independent experiments are reported in the histograms as fold increase (FI) over the control values (mean ± SEM). *Statistically significant difference from untreated cells (control), P < 0.05.

The results obtained by Western blotting showed a significant increase in the levels of pChk2 in cells treated with winter PM2.5 for 3 h (Figure 2); after 10 h of exposure, the levels of pChk2 returned to control values. Interestingly, neither the level of p53 nor its phosphorylated form were increased after PM treatments at 3 and 10 h (Figure 2); however significant increases of both forms were observed in cells exposed to the positive control topoisomerase II inhibitor etoposide.

### Characterization of the mitotic process

**Figure 3:**
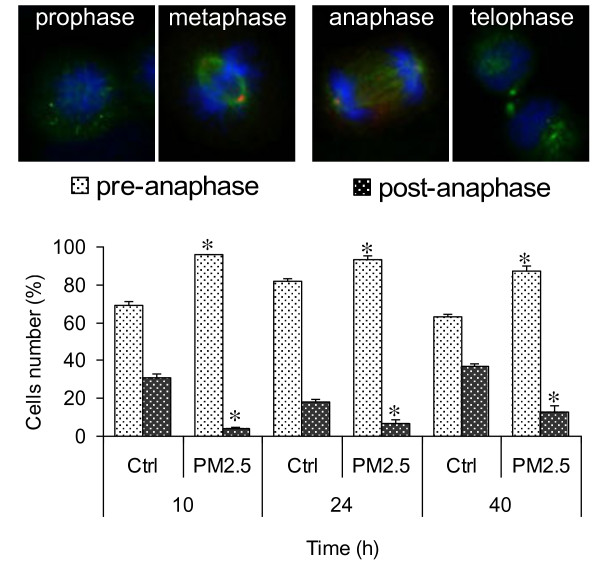
**Analysis of the mitotic phases.** BEAS-2B cells were exposed for 10, 24 and 40 h to 7.5 μg/cm^2^ of winter PM2.5, stained for DNA (blue) and β-tubulin (green) and scored as pre-anaphasic and post-anaphasic cells. The results are representative of 3 independent experiments; in each experiment 200 cells were scored. *Statistically significant difference from untreated cells (control), P < 0.05.

Cells arrested in mitosis were further characterized by fluorescence microscopy in order to determine if structural modifications of the mitotic spindle could be responsible for the observed mitotic arrest. In cultures exposed to PM2.5 for 10 h, post-anaphase (anaphase and telophase) was seen only in 4% of the mitotic cells compared to 31% in controls (Figure 3). The mitotic cells in PM exposed samples seemed to be arrested at the M/A transition point, suggesting alterations of the mitotic spindle apparatus. This imbalance among the mitosis phases was maintained at 24 and 40 h. Indeed, although the number of mitotic cells was comparable in controls and PM-treated samples, the relative count of pre- and post-anaphase cells still showed significant differences.

**Figure 4:**
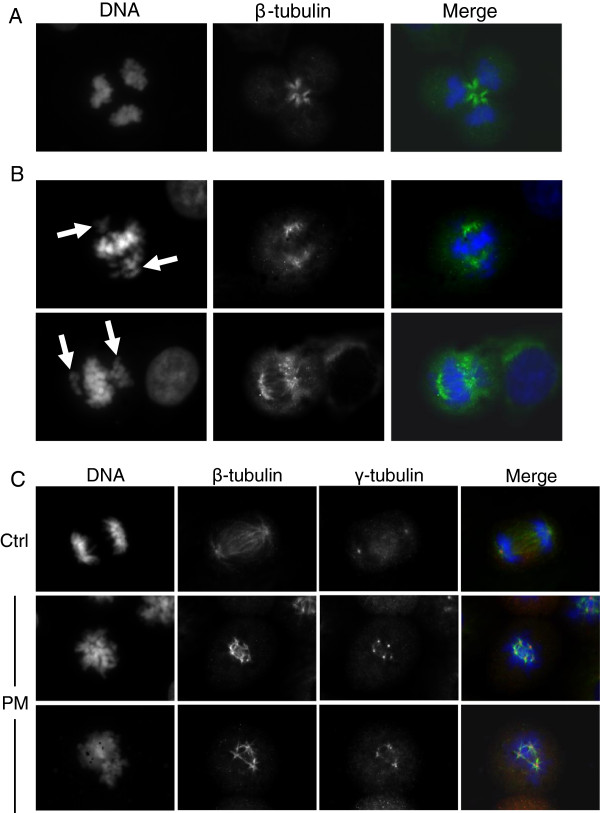
**Mitotic spindle analysis.** BEAS-2B cells were exposed for 10 h to 7.5 μg/cm^2^ of winter PM2.5: β-tubulin (green) and DNA (blue) staining evidenced tripolar mitotic cell **(A**, telophase; 8% of mitotic cells in treated samples vs. 2% in controls; statistically significant difference, P < 0.05); and bipolar incomplete spindle with groups of lagging chromosomes **(B**, arrows; 10% of mitotic cells in treated samples vs. 1% in controls; statistically significant difference, P < 0.05); γ-tubulin staining (red) showed centrosomes amplification **(C**, 6.7% of mitotic cells in treated samples vs. 2.7% in controls; statistically significant difference, P < 0.05). The results are representative of 3 independent experiments; in each experiment 300 cells were scored.

Aberrations of the mitotic spindle, represented by tripolar (Figure 4A), multipolar (Figure 4C) and incomplete (Figure 4B) spindles, were also observed. Tripolar spindles accounted for 8% of mitotic cells in PM-exposed samples compared to 2% in controls. Anaphasic and telophasic tripolar cells were also observed, suggesting that some of these cells were able to complete the mitotic division (Figure 4A). Incomplete spindles were represented by bipolar spindles with groups of lagging chromosomes (Figure 4B). This configuration occurred in approximately 10% of mitotic cells in treated samples compared to 1% of controls. Cells stained for γ-tubulin evidenced the presence of centrosome amplification associated with multipolar spindles (Figure 4C). Cells with more than 3 centrosomes represented 6.7% of mitotic cells in exposed samples compared to 2.7% in controls. Post-anaphase cells with incomplete and multipolar spindles were never observed.

**Figure 5:**
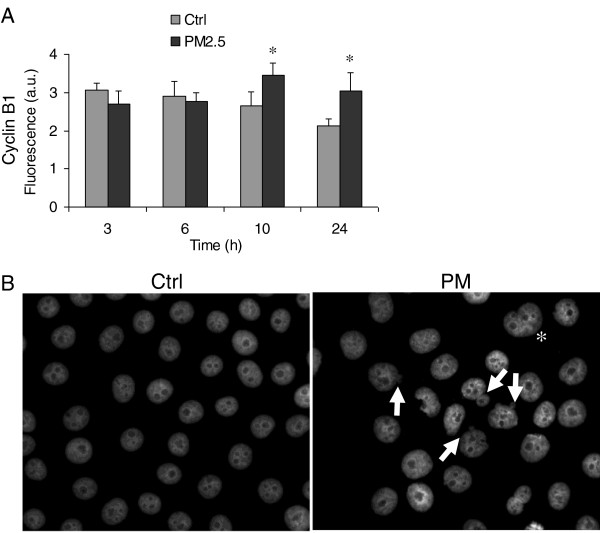
**Mitotic process analysis. (A)** Cyclin B1 expression was measured by flow cytometry in BEAS-2B cells exposed for 3, 6, 10 and 24 h to 7.5 μg/cm^2^ of winter PM2.5. The results are reported as fluorescence arbitrary units (mean ± SEM of 3 independent experiments). *Statistically significant difference from untreated cells (control), P < 0.05. **(B)** Nuclear morphology of BEAS-2B cells exposed for 24 h to 7.5 μg/cm^2^ of winter PM2.5: fluorescence microscopy illustrating micronucleated (arrows, 18.8% in treated samples vs. 3.2% in controls; statistically significant difference, P < 0.05) and binucleated (asterisk) cells. The results are representative of 3 independent experiments; in each experiment 300 cells were scored.

Since cyclin B1, associated with Cdk1, drives the progression of cells through mitosis, its level was analysed with flow cytometry. A significantly higher level of this protein was detected in cells exposed to PM for 10 and 24 h compared to controls (Figure 5A).

Finally, fluorescence microscopy analysis after 24 h of PM exposure showed cells with large abnormal nuclei and others with double-nuclei, while cells with MN were detected in 18.8% of treated samples compared to 3.2% of controls (Figure 5B). These findings suggest that the mitotic block often resulted in impaired cytokinesis and/or disturbed chromosomal separation.

### PM components responsible for G2/M delay

**Figure 6:**
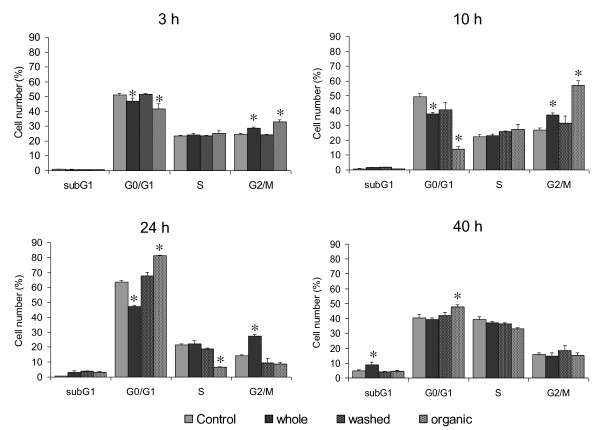
**PM fractions effect on the cell cycle.** BEAS-2B cells were exposed for 3, 10, 24 and 40 h to 7.5 μg/cm^2^ of whole-PM, the equivalent amount of organic fraction and washed particles. The results are representative of 3 independent experiments. *Statistically significant difference from untreated cells (control), P < 0.05.

To further study which PM components could be responsible for the observed effects, the organic compounds were extracted from particles; both this organic fraction and the washed particles were tested for cell cycle alterations. The G2/M increase induced after 3 and 10 h of exposure to PM organic fraction was higher than that observed in the whole-PM exposed cells, while the washed particles were ineffective (Figure 6). Interestingly after 24 h of exposure, when an increase in G2/M phase was still observed in whole-PM treated cells, an increased number of cells in G1 was seen after exposure to PM organic fraction and this increase could still be observed after 40 h of exposure. At this time point, an increased amount of cells in subG1 following exposure to whole-PM was seen (Figure 6).

### Cellular mechanisms involved in G2/M delay

**Figure 7:**
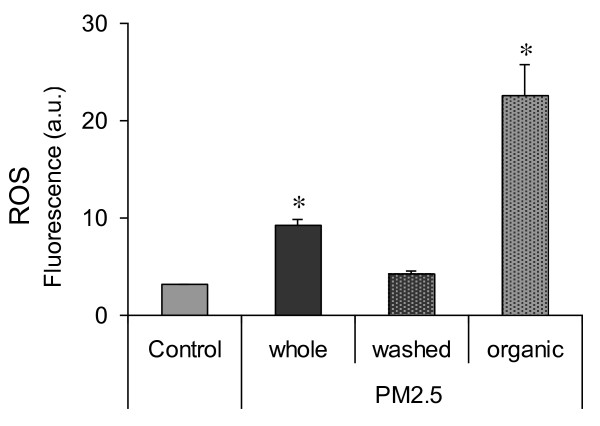
**ROS formation in BEAS**-**2B cells.** Cells were exposed for 2 h to 7.5 μg/cm^2^ of winter PM2.5 and to the equivalent amount of organic fraction and of washed particles. The results are reported as fluorescence arbitrary units (mean ± SEM of 3 independent experiments). * Statistically significant difference from untreated cells (control), P < 0.05.

ROS formation in treated BEAS-2B cells was analysed to investigate their possible involvement in the induction of the transient G2/M arrest. Notably, the PM organic fraction induced higher levels of ROS in comparison with whole-PM, resulting in a 2.4-fold increase of fluorescence intensity (22.5 a.u. in exposed cells versus 9.2 a.u. in controls). Washed particles were ineffective (Figure 7).

**Figure 8:**
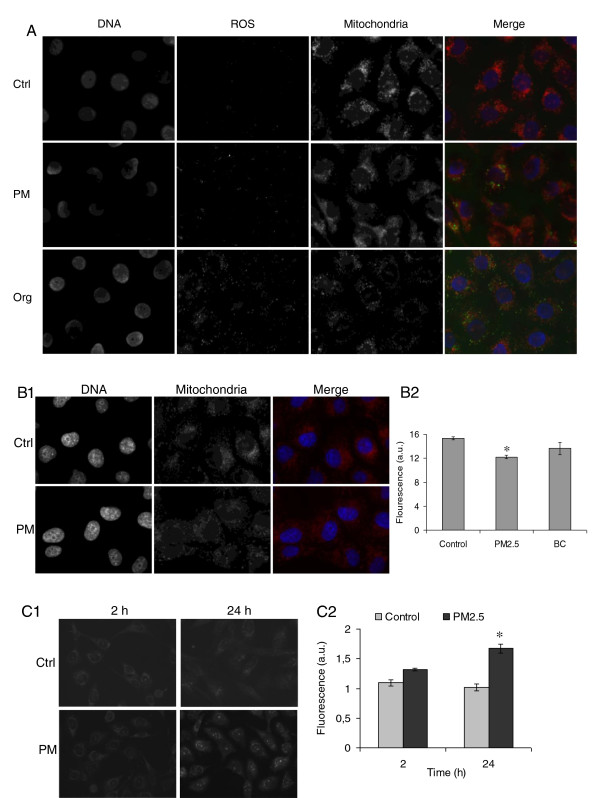
**Analysis of mitochondrial damage. (A)** ROS and mitochondria co-localization was assessed by fluorescence microscopy. BEAS-2B cells, exposed for 2 h to 7.5 μg/cm^2^ of winter PM2.5 and to its organic fraction, were stained for DNA, ROS and mitochondria. Co-localization signal of ROS and mitochondria was measured by Axiovision Rel 4.8 software as reported in materials and methods. **(B)** Mitochondrial damage in BEAS-2B cells exposed for 24 h to 7.5 μg/cm^2^ of winter PM2.5 was assessed by fluorescence microscopy **(B1)** and flow cytometry **(B2)**. Flow cytometry results are reported as fluorescence arbitrary units (mean ± SEM of 3 independent experiments). Carbon black **(CB)** was used as reference control for carbonaceous particles effects. **(C)** Mitochondrial superoxide formation in BEAS-2B cells exposed for 2 and 24 h to 7.5 μg/cm^2^ of winter PM2.5 was assessed by fluorescence microscopy **(C1)** and flow cytometry **(C2)**. Flow cytometer results are reported as fluorescence arbitrary units (mean ± SEM of 3 independent experiments). Hydrogen peroxide (1 mM) was used as positive control and reported 3.4 ± 0.11 and 12.6 ± 0.09 as fluorescence arbitrary units at 2 and 24 h, respectively. * Statistically significant difference from untreated cells (control), P < 0.05.

Mitochondria are known sources for ROS formation [32], thus their possible role in PM-induced ROS was investigated. First, the co-localization of ROS and mitochondria in cells was assessed by staining with DCFH-DA and MitoTracker, respectively. The results showed ROS as green dots spread in the cytoplasm and partially overlapping with red fluorescence of mitochondria (Figure 8A). The measurement of the fluorescent signals co-localization revealed that approximately 40-50% of ROS localized at mitochondrial level. The increase of ROS at mitochondrial level might be related to damages at the organelles’ membrane. The mitochondrial damage was then analyzed by flow cytometry. Cells treated with PM for 24 h presented a statistically significant reduction of mitochondrial fluorescence signal (MitoTracker) compared to controls (Figure 8B). In contrast, carbonaceous particles (CB) were ineffective.

To better clarify any possible role of mitochondria in ROS formation, the specific mitochondrial superoxide indicator MitoSOX was used. The results showed that mitochondrial superoxide was not significantly increased after 2 h of PM exposure (Figure 8C1 and C2). This suggests that ROS formation was not directly related to mitochondrial alteration at this time point, and the co-localization signal was due to other mechanisms occurring at/or close to the mitochondria. However, a significant increase of MitoSOX signal was measured at 24 h (Figure 8C1 and C2), when mitochondrial damage was present (Figure 8B1 and B2).

**Figure 9:**
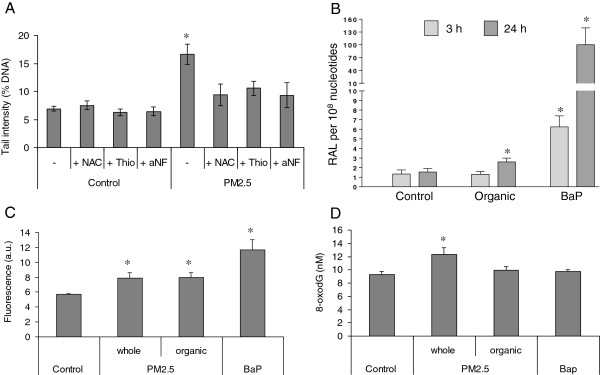
**DNA damage. (A)** SCGE assay. BEAS-2B cells were exposed to 7.5 μg/cm^2^ of winter PM2.5 for 3 h. NAC, Thio and α-NF were used to determine the role of ROS and CYP enzymes, respectively. DNA damage is expressed as % of tail intensity (% DNA in tail). **(B)** DNA adduct formation in BEAS-2B cells exposed to 7.5 μg/cm^2^ of PM organic extract and 15 μM BaP for 3 and 24 h measured by ^32^P-postlabelling. Total bulky adducts (RAL, relative adduct labelling) were measured across the DRZ, diagonal radioactive zone of the TLC autoradiogram. **(C)** γH2AX expression measured by flow cytometry. BEAS-2B cells were exposed to 7.5 μg/cm^2^ of winter PM2.5 and organic extract for 3 h, and 15 μM of BaP for 24 h. The results are reported as fluorescence arbitrary units. **(D)** Formation of 8-oxodG. BEAS-2B cells were exposed to 7.5 μg/cm^2^ of winter PM2.5 and organic extract, and to 15 μM of BaP for 3 h. The results are reported as 8-oxodG in nM. All data are expressed as mean ± SEM of 3 independent experiments. * Statistically significant difference from untreated cells (control), P < 0.05.

Since cell cycle arrest is often related to DNA damage, whole-PM2.5 and its organic extract were tested for their DNA-damaging potential. Figure 9A illustrates PM-induced DNA damage after 3 h of exposure, analysed by the SCGE-assay under alkaline conditions; a significant increase in tail intensity was present. The AhR/CYP-inhibitor α-naphthoflavone (α-NF), as well as the nucleophilic antioxidants *N*-acetylcysteine (NAC) and thiourea (Thio), significantly reduced this effect, suggesting that DNA damage might be related to the formation of reactive metabolites and ROS via the P450 system. Preliminary data with the enzyme Formamidopyrimidine DNA-glycosylase (Fpg), which converts 8-oxodG to DNA-alkali-labile sites, did not result in significant increases in DNA damage in the PM-treated samples when compared to controls (data not shown). This result is in accordance with previous findings obtained with higher PM doses after 24 h of exposure [21]. ^32^P-postlabelling analysis showed that bulky DNA adduct formation increased 1.7-fold after 24 h exposure to PM organic extract relative to controls (Figure 9B); representative autoradiograms showing DNA adduct profiles are provided as supplementary material (Additional file 1). No significant increase was observed after 3 h of exposure. Benzo[*a*]pyrene (BaP) treatment, used as positive control, resulted in significant DNA adduct formation after 3 and 24 h, confirming that BEAS-2B cells are metabolically competent to mediate CYP-catalysed PAH bioactivation.

DNA double-strand breaks (DSBs), assessed by measuring the levels of γH2AX, were increased in cells exposed for 3 h to PM2.5 and organic extract (Figure 9C); 8-oxodG was increased by winter PM2.5, while organic extract and BaP were ineffective (Figure 9D).

**Figure 10:**
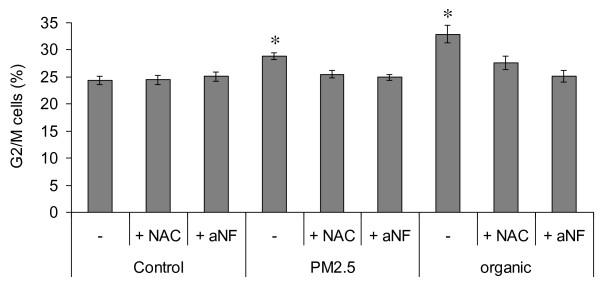
**G2**/**M cells percentage.** Cells percentage was assessed by cell cycle analysis of BEAS-2B cells exposed for 3 h to 7.5 μg/cm^2^ of whole-PM and organic fraction. NAC and α-NF were used to determine the role of ROS and CYP enzymes, respectively. * Statistically significant difference from control, P < 0.05. The results are representative of 3 independent experiments.

α-NF and NAC completely abolished the G2/M-accumulation visible after exposure to PM or its organic fraction (Figure 10), confirming that ROS and P450-formed reactive metabolites of the organic fraction are responsible for the cell cycle delay.

## Discussion

In the present study we showed that 7.5 μg/cm^2^ of a well-characterized urban fine PM (Milan winter PM2.5) caused alterations in different phases of the cell cycle, resulting in apoptotic cell death, tetraploid G1 cells (binucleated) and cells with MN.

PM exposure has previously been reported to result in an accumulation of cells at various cell cycle phases [20,22,23]. Besides PM characteristics and dose, time of analysis and the specific cell line used may also influence the results obtained [23,33]. We have previously reported that 25 μg/cm^2^ of Milan winter PM2.5 induced mitotic arrest in BEAS-2B cells after 20 h of exposure which later resulted in mitotic cell death [21]. Here we investigated the *in vitro* effects of a PM-dose which is among the lowest reported in literature to give biological effects, in an effort to approach environmental human exposure levels. Using this dose, the various phases of the cell cycle were differently affected and little mitotic apoptosis was observed. As results on cell cycle distribution are highly dependent on the time of the analysis, the cell cycle progression has been followed at different time points. A significant increase of cells in G2/M phases already occurred after 3 h of exposure. The G2/M increase was sustained up to 24 h, but it consisted of alterations at three different phases of the cell cycle progression. The combined use of flow cytometry and fluorescence microscopy revealed an early (after 3 h) delay in the G2 phase. This was followed by an increased number of cells in mitosis (after 10 h). Finally, cytokinesis was affected, because an increased number of non-mitotic tetraploid (4 N) G1 cells was seen after 24 h. The increase of cells in the subG1 region suggests that part of the cells affected by PM treatment die through apoptosis at 40 h.

The cell cycle delay has often been linked to DNA damage and the DNA damage response [20,23,34]. The G2/M transition checkpoint is a non-genomic and rapid-response system activated by DNA damage response [24]. The rapid G2 block is primarily induced in a transient mode and requires p53 transcriptional activity to ultimately produce a sustained block [24,27]. Transient or sustained by p53, the checkpoint protein kinase Chk2 is a pivotal messenger of this system. In the present study we observed a significant increase in the level of the active phosphorylated form of Chk2 (pChk2) in cells treated with winter PM2.5 for 3 h, which is in line with the accumulation in G2 phase reported. The levels of pChk2 decrease to control values after 10 h of exposure, suggesting that the cells have overcome the G2 arrest and have entered mitosis. Accordingly, the levels of p53 and pp53 appear not to be affected by PM treatment at 3 and 10 h; these data confirm that cells exposed to PM were arrested transiently in G2 by a p53-independent pathway at 3 h of exposure and then escape from G2 into mitosis after 10 h.

When studying DNA damage and DNA damage responses *in vitro* it is essential to avoid cell lines with *TP53* mutations, as the loss of p53 activity is linked to defects in cell cycle control and apoptosis after DNA damage [35]. Here we used BEAS-2B cells, which are reported to have normal p53 activity, and for this reason have been widely used to study cell cycle alterations [36] and mechanisms involved in PM-induced toxicity [37,38]. Nevertheless, it should be noted that this cell line is SV 40-transformed, thus these effects should be further explored in primary human lung epithelial cells and/or *in vivo*.

The alterations of the cell cycle may not only depend on DNA damage but also on damages to other macromolecules, as well as on changes in protein phosphorylation and ion concentrations [24]. As shown in the present study, the various cell cycle steps affected in PM2.5-exposed cells suggest that several types of initial damage might be involved. The mitotic arrest was characterized by disequilibrium in the different mitotic phases (higher incidence of pro- and metaphase cells versus ana- and telophase ones) suggesting possible structural dysfunctions of microtubules (MT) and of mitotic spindle assembly. Furthermore, mitotic cells presented various aberrations of the mitotic apparatus, including tripolar, multipolar and incomplete spindles. Moreover, γ-tubulin staining showed centrosomes amplification. Similar spindle aberrations have been reported in Chinese hamster fibroblasts after exposure to PM10 [39] and in our previous study, where preliminary results showed the presence of tripolar cells [21]. These findings indicate that PM may act as spindle poison, directly perturbing microtubules dynamics, and suggest the activation of the spindle assembly checkpoint (SAC) as a mechanism for the M/A delay. Indeed, centrosomes amplification and increased number of spindle poles are known to cause a delay in the anaphase onset through SAC activation [40]. Further, SAC can also be activated by the presence of incomplete bipolar spindles with lagging chromosomes, similar to the ones we found. Pole-associated chromosomes are a regular transient feature of astral spindle assembly, when an initial monotelic attachment brings the chromosomes towards the centrosomes. Under normal conditions this feature should be rapidly corrected by an Aurora-B kinase-based mechanism [24]. The presence of a high percentage of cells with pole-associated chromosomes (10% in PM-treated samples) suggests a delay in the rearrangement of this attachment.

After exposure to PM for 24 h the number of cells was slightly reduced relative to controls, without significant levels of mitotic-apoptosis. However, an increased number of non-mitotic cells with double amount of DNA (4 N), large or double nuclei, and cells with micronuclei (MN) were present, suggesting that cells, when arrested in mitosis, did not always complete cytokinesis. It is well known that cells arrested by SAC at the M/A transition point can exit mitosis without proper segregation of chromosomes and cytokinesis, if the damages are not properly corrected within a certain period of time. This process (called mitotic slippage) gives rise to cells with large or double nuclei (4 N, G1) and with multiple micronuclei [29], as we found. In agreement with the literature [30], cells with amplified centrosomes, forming tripolar mitotic spindles, seemed to go through karyokinesis, as tripolar cells in anaphase and telophase were frequently observed. These cells might contribute to the increased subG1 peak reported after 40 h of exposure, which can be only partly explained by the increase of apoptosis observed at this time point. In contrast, cells with more than three poles were never found in anaphase and telophase, suggesting that they failed the cytokinesis, resulting in binucleated or micronucleated cells.

Cells exposed for 24 h to PM also presented high levels of cyclin B protein. This further supports the hypothesis of SAC activation, as SAC inhibits the anaphase-promoting complex (APC)-dependent degradation of cyclin B. Moreover it has been demonstrated that cyclin B degradation not only is required for the transition to anaphase, but also for the onset of cytokinesis in *Drosophila*[41]. Interestingly, Burns et al. [42] found high levels of cyclin B1 in 4 N cells treated with nocodazole and paclitaxel. On the other hand, Brito and Rieder [43] reported that cyclin B degradation is required for mitotic slippage; thus the role of cyclin B in this event is still a matter of debate.

The results obtained from the various PM fractions (organic fraction versus inorganic and carbonaceous particles) showed that the organic components of Milan winter PM2.5 are very important for the effects on the cell cycle, as particles deprived of these compounds were ineffective. This observation is in line with previous results showing that Milan summer PM2.5, with low quantity of PAHs, had no effect on the mitotic progression [44]. Accordingly, other data in the literature [45-47] describe the role of PM organic compounds in inducing toxicity. In most of these studies [48,49], the high PAHs content has been associated with high genotoxicity, oxidative stress, and mitochondrial and cytoskeletal dysfunctions. Möller and colleagues [50] reported effects on phagocytosis, phagosome transport mechanisms and cytoskeletal integrity. PAHs-rich PM0.2, produced by combustion of solid fuels, induced G2/M arrest in macrophages [23], while organic extracts from PM2.5 and PM10 arrested the cell cycle of different human cell lines in G0/G1 [22,51]. Several PAHs are able to alter the cell cycle in various ways; dibenzo[*a*,*l*]pyrene induces G2/M arrest in human mammary carcinoma MCF-7 cells [52], while it delays HEL fibroblasts in the S phase [53]. Similarly, exposure to BaP leads to S phase accumulation in human hepatocarcinoma HepG2 and MCF-7 cells [54]. Moreover, recent results have shown that the cell cycle status can impact on BaP metabolism and DNA damage [55]. Thus, how PAHs adsorbed on PM may affect the cell cycle depends on the specific compounds present and the cells’ metabolic capacity. The compounds’ bioavailability is also of importance, which was demonstrated in the present study by the higher potential of the PM organic fraction in comparison with the whole-PM to induce ROS formation. On the other hand, the whole-PM longer sustained the cellular arrest in G2/M when compared to the organic fraction, and induced oxidative DNA damage. Thus, the localization of PAHs on the particles is probably of importance for some of the PM-induced effects. However, a role for other components cannot be excluded. These could be some metals in the water soluble PM fractions, which have been shown to alter mitosis progression [56,57].

The organic fraction seemed to be responsible for the increase of ROS observed at 2 h of exposure. ROS formation after PM exposure is associated with significant cell effects such as mitochondrial damage, increased production of cytokines and chemokines, as well as DNA damage [2,58,59]. Moreover, high levels of oxidants determine perturbation of the mitochondrial permeability and a disruption of electron transfer chain resulting in cellular apoptosis or necrosis [58]. Mitochondria have been indicated as the main source of ROS generation in rat alveolar type II and human lung adenocarcinoma A549 cells exposed to a high dose of PM2.5 (50 μg/cm^2^) [32]. However in this study, after exposure to 7.5 μg/cm^2^, only 40-50% of total ROS were localized at the mitochondria, while the rest of ROS were located in the cytoplasm. Moreover, the absence of mitochondrial superoxide formation indicated that mitochondria are not significantly involved in ROS production at 2 h. Considering these results, it is likely that the organic fraction is responsible for PM-induced ROS through P450-mediated metabolic activation of various PAHs and oxo-PAHs. The co-localization of ROS signal and mitochondria might be due to CYP enzymes, which have been recently reported to have also mitochondrial localization [60]. Still, the contribution of other pathways (such as AKR or NADPH oxidase) cannot be excluded [61] and should be further investigated.

As mitochondrial superoxide formation was found at 24 h, this effect is likely secondary to ROS formation, and may be caused by the observed mitochondrial damage.

The results in this study show that PM was able to induce DNA damage as determined by comet assay, measuring strand breaks and alkali-labile sites. The AhR-response has previously been found to be of major importance in explaining the toxicity of various PM [21,62,63] and of its organic fraction [64]. In accordance with this, antioxidants NAC and Thio, and the AhR/CYP enzymes inhibitor α-NF reduced the PM-induced DNA damage, as well as the G2 increase occurring at 3 h of exposure. These findings suggest that these effects were related to ROS and/or other reactive metabolites formed by AhR/CYP enzymes.

ROS-induced DNA damage includes various oxidative DNA base modifications as well as single and double strand breaks (SSBs and DSBs) [65,66], while the reactive PAHs intermediates might also induce bulky DNA adducts [62,67]. A further characterization of PM-induced DNA damage by ^32^P-postlabelling showed that the PM organic fraction induced higher bulky DNA adduct levels after 24 h of exposure, while no difference was seen after 3 h. Similar results following PM exposure have been reported by others [15,62]. PAHs which form DNA adducts often require a two-steps activation [67], which might undergo competitive inhibition by non-genotoxic PAHs present in the PM complex mixture [68]. Thus, the primary DNA damage detected by the comet assay might be those induced by organics and PAHs needing only one-step activation, such as nitro- and oxo-PAH.

Although the comet assay with Fpg was negative, the levels of 8-oxodG and γH2AX measured by immunostaining increased after 3 h of PM exposure, suggesting the presence of oxidative DNA damage and DSBs. A similar lack of effect of comet assay with Fpg, despite positive immunostaining, have previously been reported [21] and is probably due to an artefact; various micro and nanoparticles have been reported to interact with Fpg, decreasing the sensitivity of the assay [69], and PM may have similar effects.

Interestingly, 8-oxodG was increased by whole-PM but not by its organic extract, suggesting a more direct interaction of some PM component (including both metals and various PAHs) with the DNA in the nucleus [70-72]. It is known that 8-oxodG is induced by singlet oxygen and hydroxyl radical which, due to their high reactivity, will only react with DNA when generated in direct proximity [65]. Thus, our results suggest that ROS formed in the cytosol when exposed to the organic fraction will not interact with the cellular DNA. Previous data in our laboratory indicated that PM may be in close contact with the chromosomes [21], but the current data is not conclusive and this potential nuclear localization of PM would require further investigations.

In conclusion, the dose used in the present study is among the lowest reported to have biological effects *in vitro*[73]. Our study shows that this low dose of winter PM2.5 induces an early G2 arrest followed by an arrest in M/A with a subsequent inhibition of cytokinesis and an increased formation of cells with double nuclei and MN. These effects are associated with a rapid DNA damage response and the formation of mitotic spindle aberrations. The early DNA damage and G2/M accumulation have been related to the formation of reactive electrophilic/radical metabolites via a P450-depending reaction. However, PM2.5 apparently also has spindle poison properties which contribute to the induction of the M/A arrest. The characterization of the process leading to double nuclei and MN in PM-exposed cells is of great importance, giving a possible explanation for PM-induced chromosomal aberrations. Such events could be central when explaining the increased lung cancer incidence associated with PM2.5 and deserve further investigations.

## Materials and methods

### PM collection and preparation

PM samples were collected during winter 2009/2010 at Torre Sarca, a site of Milan urban background for atmospheric pollution. Milan winter PM2.5 ambient concentration is 50 μg/m^3^ ± 29 (mean ± SD). Samplings were performed on Teflon filters by a low volume gravimetric sampler (EU system, FAI Instruments, Rome, Italy); on average the particles mass was 1.5 mg per filter after 24 h of sampling. Filters were replaced every 24 h and then they all were stored in one pool representative of the winter PM. Particles were extracted as previously described [74]. Briefly, filters were detached from plastic holders and, after immersion in 2 ml of sterile water, underwent four cycles of 20 min each in an ultrasound bath (SONICA, Soltec). The extraction water was replaced every sonication cycle, and the volumes obtained from the four cycles were put together to obtain a homogeneous sample. Particle suspensions were dried in a desiccator (72 h), weighed and stored at -20°C, and the resulting pellets were re-suspended in sterile water (2 μg/μl) just prior to use.

This standardized procedure for ambient particulates does not modify the natural state of particles aggregates. The extraction efficiency, *i.e*. PM mass extracted compared to the PM total mass on filters, has been found to be approximately 75% regardless of the dimension, origin or chemical composition of the particles (similar efficiency for PM1, PM2.5 and PM10, sampled in summer or winter). These observations assure the similarity between the extracted particles and the original ones.

As an additional check of the method, solutions have been produced in the same way from unloaded teflon filters and used to treat the cells; various toxicological tests were performed (viability, release of inflammatory proteins, cell cycle alterations and ROS formation) and no effects have been observed in comparison to untreated control cells.

PM2.5 organic extract was obtained by re-suspending particle pellets in acetonitrile (Sigma-Aldrich), according to the procedure used for the chemical characterization of PM. The extraction efficiency has been evaluated and recoveries were over 65% for all the analyzed PAHs [75]. After centrifugation (15 min, 12000 rpm, 4°C), supernatant and pellet were separated and dried in a desiccator. The organic fraction, obtained from the supernatant, was dissolved in DMSO (10 μg/μl of original pellet weight), while washed particles were re-suspended in sterile water (2 μg/μl of original pellet weight).

The chemical and morphological characterization of the PM used has been previously reported [74-76]. Briefly, suspensions obtained from atmospheric samples were analysed by transmission electron microscopy. The winter PM2.5 appeared as aggregates of small, round-shaped particles, and the particle size distribution confirmed that few particles exceeded 1 μm in diameter [74]. Analyses by IC, TOT, ICP-MS and GC-MS evidenced that particles were mainly composed of water-soluble inorganic ions (NH_4_^+^, NO_3_^-^ and SO_4_^2-^), organic and elemental carbon, and elements. A high PAH concentration (0.06% of the total PM mass) was measured, and the most abundant elements were Fe, Zn and Al.

### Cell culture and exposure

The human bronchial epithelial cell line BEAS-2B (SV40 hybrid, Ad12SV40, transformed) was purchased from the European Collection of Cell Cultures (ECACC, Salisbury, UK). Cells were maintained in LHC-9 medium at 37°C with 5% of CO_2_, split every three days and the medium was changed the day after. For experiments, cells were seeded at a concentration of 80,000 cells/well in 6-well plates, or 1 × 10^6^ cells in Petri dishes (Ø = 10 cm), and after two days treated with 7.5 μg/cm^2^ of winter PM2.5 or the equivalent amount of organic extract/washed particles. The exposure dose used was selected on the basis of a previous study, choosing a low effective dose [21]. The cellular responses were examined after 1, 3, 6, 10, 24 and 40 h of exposure and the results compared to those of untreated cells (control). Cells were pre-incubated for 1 h with antioxidants, NAC (10 mM) or Thio (100 μM), or the CYP/AhR inhibitor α-NF (10 μM), before exposure to particles. CB (7.5 μg/cm^2^; Sigma-Aldrich, Italy) was used as a reference carbonaceous material. Hydrogen peroxide (H_2_O_2_, 1 mM final concentration), topoisomerase II inhibitor etoposide (Etop, 250 nM) and benzo[a]pyrene (BaP, 15 μM) were used as positive controls for mitochondrial superoxide formation, p53/pp53 activation and DNA adduct formation, respectively.

### Flow cytometry

#### Cell cycle analysis

The cell cycle after exposure to PM, PM-extracts, or washed PM was analyzed at different time points by flow cytometry. Briefly, cells were harvested, fixed in 70% ethanol at -20°C and stored until analysis. After centrifugation, cells were resuspended in PBS with 20 μg/ml RNase DNase-free (Sigma-Aldrich, Italy) and incubated at 37°C for 30 min. Propidium iodide (PI) was added and fluorescence was measured by the flow cytometer EPICS XL-MCL (Beckman-Coulter) using a 575 nm band pass filter. Data were analyzed using the EXPO32 ADC software (Beckman-Coulter).

#### Cyclin B1 expression

Cyclin B1 levels were assessed by flow cytometry. Cells were harvested, fixed with 1% paraformaldehyde on ice for 15 min, resuspended in cold methanol 90% and stored overnight at -80°C. After centrifugation, cells were washed once in PBS/0.5% BSA and incubated with primary antibody (Cyclin B1, 1:100 dilution; Cell Signaling) in PBS/0.5% BSA/0.2% Triton X-100 overnight at 4°C. Alexafluor-488 secondary antibody (1:500; Invitrogen) was incubated for 1 h at room temperature. Finally, cells were washed once in PBS/0.5% BSA, resuspended in PBS and analyzed by flow cytometry. Fluorescence of 10,000 events was detected using a 525 nm band pass filter.

#### ROS formation

ROS was measured by the fluorescent probe 2’7’-dichlorodihydrofluorescein diacetate (DCFH-DA; Life Science Technologies, Italy). Cells were incubated at 37°C with DCFH-DA (5 μM) in PBS for 20 min, washed in PBS and treated with PM, organic extract or washed particles for 45 or 120 min, harvested and suspended in PBS. The ROS-linked fluorescence was quantified by flow cytometry using a 525 nm band pass filter. The auto-fluorescence of cells, PM and PM organic extract was assessed analysing the signal from negative controls (samples not stained with DCFH-DA). These values were then subtracted from the values to DCFH-DA stained samples.

#### Mitochondrial signal

MitoTracker Red CMXRos (Invitrogen) was used to measure mitochondrial integrity since the fluorescence signal of this dye is dependent upon membrane potential. Thus, a reduction of MitoTracker fluorescence is considered an indication of decreased mitochondrial membrane potential. BEAS-2B cells exposed for 24 h to winter PM2.5 and CB (7.5 μg/cm^2^) were harvested, stained with MitoTracker (30 min, 50 nM) and fluorescence of 10,000 events was detected using 575 nm band pass filter on the flow cytometer. CB was used to exclude the possibility that the eventual mitochondrial signal reduction may be due to an interaction of the particles with the probe.

MitoSOX Red mitochondrial superoxide indicator (Invitrogen) was used to investigate the role of mitochondria in ROS formation, since this dye selectively detects the superoxide formation in the mitochondria. BEAS-2B cells were exposed for 2 and 24 h to winter PM2.5 (7.5 μg/cm^2^) and H_2_O_2_ (positive control, 1 mM). At the end of the treatment 2 μM MitoSOX Red working solution was freshly prepared in HBSS/Ca/Mg and incubated with the cells for 15 minutes at 37°C, in the dark. Then, cells were harvested and the fluorescence of 10,000 events was detected using a 575 nm band pass filter on the flow cytometer.

### Fluorescence microscopy

#### Immunocytochemistry

Cells were stained for β-tubulin and γ-tubulin to observe mitotic microtubules (MTs) and centrosomes, respectively. Cells for immunocytochemical detection of proteins were prepared following common fluorescence microscopy techniques. Briefly, cells grown on cover slips were treated with PM as described above, washed in PBS and fixed with 1% paraformaldehyde for 15 min on ice. Permeabilization and blocking were performed in PBS/0.5% BSA/0.2% Triton X-100 for 15 min at room temperature. Cells were then immunocytochemically labelled with primary antibodies in PBS/0.5% BSA/0.2% Triton X-100 overnight at 4°C (β-tubulin 1:200 dilution; γ-tubulin 1:1000; Cell Signaling). Appropriate Alexafluor secondary antibodies (1:500 dilution; Invitrogen) were incubated for 1 h at room temperature and cells’ DNA counterstained with DAPI. Slides were observed under a fluorescence microscope (AxioObserver, Zeiss Germany) and digital images were taken.

The percentage of mitotic and apoptotic cells was assessed by fluorescence microscopy in samples exposed to PM for 3, 10 and 24 h. According to nuclear morphology, 500 cells per samples were scored as interphasic (uncondensed chromatin), mitotic (condensed chromosomes) or apoptotic (typical highly condensed and fragmented nuclei) cells. Mitotic cells (200 cells per sample) were analysed to assess the mitotic phase; according to arrangement of chromosomes and mitotic spindle, cells were scored as pre-anaphasic (pro- and metaphase) or post-anaphasic (ana- and telophase) cells. After 10 h, 300 cells per sample were scored to further describe the mitotic process, analysing the presence of tripolar and multipolar mitotic cells, and bipolar cells with incomplete spindles and groups of lagging chromosomes. After 24 h, nuclear morphology of 300 cells per sample was observed to investigate the presence of micronuclei (MN) and double nuclei.

#### Fluorescence microscopy of living cells

ROS formation and effects on mitochondria were analysed in living cells using DCFH-DA, MitoTracker and MitoSOX dyes. ROS and mitochondria co-localization was investigated after 2 h of PM treatment. Cells grown on cover slips were first incubated at 37°C with 5 μM of DCFH-DA in PBS for 20 min, then exposed to PM and finally stained with MitoTracker for 30 min and counterstained with DAPI. Slides were observed under a fluorescence microscope (AxioObserver, Zeiss), digital images were taken with a final magnification of 630× (10x ocular and 63× objective lens, immersion oil) and co-localization signal was quantified with Axiovision Rel 4.8 co-localization dedicated software (Zeiss). Images of mitochondria stained with MitoTracker were also taken after 24 h of treatment with PM, to investigate possible secondary effects. Finally, the formation of mitochondrial superoxide was examined by staining the cells with MitoSOX. Briefly, after 2 and 24 h of PM treatment, cells grown on cover slips were loaded with 2 μM MitoSOX working solution for 15 min at 37°C, in the dark. Then, cells were washed in HBSS/Ca/Mg and fixed with 3% paraformaldehyde for 15 min. Digital images were taken by a fluorescence microscope with a final magnification of 630× (AxioObserver, Zeiss).

### Western blotting

The expression levels of p53 and Chk2, and of their active phosphorylated forms pp53 and pChk2, were analyzed by Western blotting to assess their involvement in cell cycle regulation. After 3 and 10 h of exposure to winter PM2.5, cells were collected, washed in PBS and stored overnight at -80°C. Cells were lysed in RIPA buffer (50 mM Tris-HCl pH 8; 150 mM NaCl; 1% NP-40; 0.5% sodium deoxycholate; 0.1% SDS), sonicated three times for 30 sec on ice and finally homogenised using a syringe needle. Cell lysates were then separated by SDS-PAGE on 10% gels and transferred to nitrocellulose membranes. Blots were incubated with appropriate antibodies (p53, phospho-p53 Ser15, Chk2, phospho-Chk2 Thr68, Cell Signaling Technology, dilution 1:1000; actin, Sigma Aldrich, dilution 1:2000) overnight at 4°C. After washes, the membranes were incubated with HRP-linked secondary antibodies (anti-rabbit IgG, Cell Signaling Technology, 1:2000; anti-mouse IgG, Sigma Aldrich, 1:80000) and subsequently incubated with Chemiluminescent Peroxidase Substrate (Sigma Aldrich) for detection. Digital images were taken by a luminescence reader (UVP) and densitometry analysis was performed with dedicated software (Launch VisionWorks LS). Data were normalized to the actin content and expressed as fold increase (FI) over control.

### DNA damage

#### Single cell gel electrophoresis (SCGE)

After 1 h exposure to antioxidants and inhibitors and 3 h exposure to PM, media were removed and cells trypsinized and resuspended at 1 million cells/ml in PBS. Samples were analysed for DNA strand breaks and alkali-labile sites using the comet assay. Cells dissolved in 0.68% LMP agarose (Gibco BRL 5517US) in PBS with 10 mM EDTA, pH 7.4, were moulded onto GelBond films attached to plastic frames to facilitate subsequent steps. Films underwent lysis (2.5 M NaCl, 0.1 mM KCl, 0.5 mM EDTA, pH 8) overnight at 4°C, and then were transferred to cold electrophoresis solution (0.3 M NaOH, 0.1 M EDTA, >pH 13.2) for 40 min at 4°C for DNA unwinding. After electrophoresis (~0.8 V/cm, 30 min, pH 13.2) and neutralisation, films were fixed in ethanol and dried. Rehydrated samples were stained with SybrGold (0.08 μl/ml in TE-buffer pH 7.4, 20 min) and scored with Perceptives Comet IV software. The level of DNA damage was expressed as tail intensity, *i.e*. percent fluorescence in the comet tail, relative to the comet total fluorescence.

#### ^32^P-postlabelling

DNA adducts were measured by the thin-layer chromatography (TLC) ^32^P-postlabelling method using the nuclease P1 digestion enrichment version of the assay [77]. After 3 and 24 h exposure to PM organic extract and BaP (15 μM), cells were washed in PBS, scraped and stored at -80°C. DNA was isolated from cells by a standard phenol extraction method and DNA samples were analysed as described [78,79] with minor modifications. Briefly, DNA (4 μg) was digested with micrococcal nuclease (288 mU; Sigma, UK) and spleen phosphodiestase (1.2 mU; MP Biomedicals, UK), enriched and labelled as reported. Solvent conditions for the resolution of ^32^P-labelled adducts on polyethylenimine-cellulose TLC (Macherery-Nagel, Germany) were: D1, 1.0 M sodium phosphate, pH 6.0; D3, 4 M lithium-formate, 7 M urea, pH3.5; D4, 0.8 M lithium chloride, 0.5 Tris, 8.5 M urea, pH 8.0. After chromatography, TLC sheets were scanned using a Packard Instant Imager (Dowers Grove, IL, USA) and DNA adduct levels (RAL, relative adduct labelling) were calculated from the adduct counts per minute (cpm), the specific activity of [γ-^32^P]ATP (Hartmann-Analytic, Germany) and the amount of DNA (pmol of DNA-P) used. As in prior studies, total DNA adduct levels were measured in the diagonal radioactive zone (DRZ) area of the TLC plates and were considered representative of PAH-DNA and other aromatic/hydrophobic adducts resistant to nuclease P1 digestion [78,79]. The method provides a summary measure of a complex mixture of adducts present in the postlabelling chromatograms. Results were expressed as DNA adducts/10^8^ nucleotides. Each DNA sample was determined by two independent ^32^P-postlabelling analyses. An external BaP-diol-expoxide (BPDE)-DNA standard was employed for identification of adducts in experimental samples.

#### γH2AX

In order to further investigate DNA damage, γH2AX was assayed by flow cytometry as a marker of oxidative DSBs. After 3 h of exposure to PM, organic extract and BaP, cells were harvested, fixed with 1% paraformaldehyde on ice for 15 min, and stored in cold 90% methanol at -80°C until analysis. Cells were then washed in PBS/0.5% BSA and incubated 4 h with Alexafluor-488 conjugated γH2AX antibody (1:100 dilution, Cell Signaling) in PBS/0.5% BSA/0.2% Triton X-100 at room temperature. Finally, cells were washed and resuspended in PBS and analysed on the Beckman Coulter EPICS XL-MCL flow cytometer. Fluorescence of 10,000 events was detected using 525 nm band pass filter.

#### 8-oxodG

The formation of 8-oxodG was investigated as a marker of oxidative DNA damage and oxidative stress, using an 8-oxodG ELISA kit (Trevigen). After 3 h of exposure to PM, organic extract and BaP, cells were trypsinized, washed with PBS and stored at -80°C. DNA was extracted using a commercial kit according to the manufacturers’ instructions (Qiagen, Flexigen Kit). DNA samples were supplemented with cations and DNase I in proper quantities and incubated for 1 h at 37°C. Alkaline phosphatase was then added, and samples were further incubated for 1 h at 37°C. DNA samples and 8-oxodG standards were mixed with anti-8-oxodG monoclonal solution in a 96-well plate and incubated for 1 h at 25°C. Wells were washed with PBS/0.1% Tween 20 and goat anti-mouse IgG-HRP conjugate antibody was added and incubated for another hour. Finally, TACSSapphire was added for 15 minutes at 25°C. The reaction was stopped by 0.2 M HCl and the absorbance was immediately read by a multiplate reader at 450 nm.

### Statistical analyses

Statistical differences between samples were tested with one-way ANOVA and post hoc comparisons performed with Dunnett’s method, by using SigmaStat 3.1 software. For the analysis of the mitotic cells and of p53/pp53 and Chk2/pChk2 paired *t*-test was used. Statistical differences were considered to be significant at the 95% level (p < 0.05).

## Abbreviations

NAC: *N*-acetyl cysteine; Thio: Thiourea; α-NF: α-naphthoflavone; M/A: Metaphase/anaphase transition point; ROS: Reactive oxygen species; PM: Particulate matter; CB: Carbon black; MN: Micronuclei; Etop: Etoposide.

## Competing interests

The authors declare that they have no competing interests.

## Authors’ contributions

EL performed the experiments and manuscript writing. KBG supervised the comet assay procedure and results analysis. VMA and JEK conducted the ^32^P-postlabelling assay. VMA, JEK and MC helped with the writing of the manuscript. JAH and MG defined the experimental plan, supervised all the experiments, interpreted the results and contributed to writing the manuscript. All authors read and approved the final manuscript.

## Supplementary Material

Additional file 1 **DNA adduct formation in BEAS-2B cells exposed to 7.5 μg/cm**^**2**^** of PM organic extract and 15 μM BaP for 3 and 24 h.** Representative autoradiograms showing DNA adduct profiles. The origin, at the bottom left-hand corner, was cut off before exposure. The arrow shows 10-(deoxyguanosin-*N*^2^-yl)-7,8,9-trihydroxy-7,8,9,10-tetrahydro-BaP (dG-*N*^2^-BPDE). Click here for file
