# Supplementary material for: Cell cycle alterations induced by urban PM2.5 in bronchial epithelial cells: characterization of the process and possible mechanisms involved
Source: Part Fibre Toxicol. 2013 Dec 19;10:63. doi: 10.1186/1743-8977-10-63 (PMC3878321; doi:10.1186/1743-8977-10-63)
Supplement: Additional file 1 — DNA adduct formation in BEAS-2B cells exposed to 7.5 μg/cm 2 of PM organic extract and 15 μM BaP for 3 and 24 h. Representative autoradiograms showing DNA adduct profiles. The origin, at the bottom left-hand corner, was cut off before exposure. The arrow shows 10-(deoxyguanosin-N2-yl)-7,8,9-trihydroxy-7,8,9,10-tetrahydro-BaP (dG-N2-BPDE). [file 1743-8977-10-63-S1.doc]

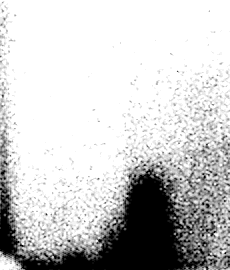

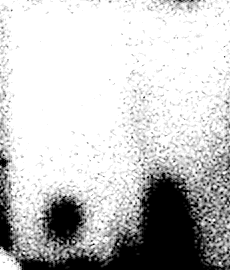

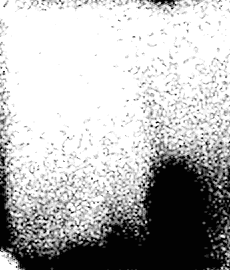

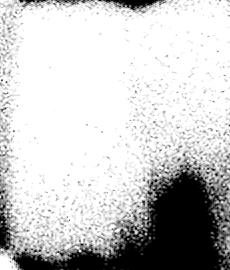

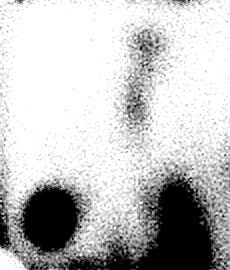


Ctrl


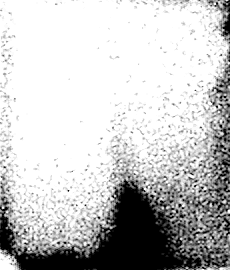


Org

BaP

3 hr

24 hr

DRZ

DRZ

DRZ

DRZ

DRZ

DRZ

DNA adduct formation in BEAS-2B cells exposed to 7.5 μg/cm^2^ of PM organic extract and 15 μM BaP for 3 and 24 h. Representative autoradiograms showing DNA adduct profiles. The origin, at the bottom left-hand corner, was cut off before exposure. The arrow shows 10-(deoxyguanosin-*N*^2^-yl)-7,8,9-trihydroxy-7,8,9,10-tetrahydro-BaP (dG-*N*^2^-BPDE).
